# Immune-Therapy-Related Toxicity Events and Dramatic Remission After a Single Dose of Pembrolizumab Treatment in Metastatic Thymoma: A Case Report

**DOI:** 10.3389/fimmu.2021.621858

**Published:** 2021-04-15

**Authors:** Li Shen, Haiyan Chen, Qichun Wei

**Affiliations:** Department of Radiation Oncology, Key Laboratory of Cancer Prevention and Intervention, Ministry of Education, The Second Affiliated Hospital of Zhejiang University School of Medicine, Hangzhou, China

**Keywords:** thymoma, pembrolizumab, immune-therapy–related toxicity events, case report, immunotherapy

## Abstract

Immune checkpoint inhibitor therapy has become a promising option for the treatment of late-stage thymic epithelial tumors. In this manuscript, we presented a patient with metastatic thymoma administrated of anti-programmed cell death protein 1 (PD-1) antibody pembrolizumab. Although the patient underwent a rapid and dramatic response to one dose of pembrolizumab, she suffered a storm of immune-therapy related toxicity events (irAEs), including liver and kidney dysfunction, hypothyroidism and myocarditis. We didn’t observe >grade 3 irAEs, and proceed with pembrolizumab therapy after the function recovered. Although no guidelines recommend dose reduction of immunotherapy re-treating following initial irAEs, we optimize dose of pembrolizumab to minimize the irAEs induced by PD-1 antibody while maintaining clinical effectiveness. Excitingly, we observe remarkable tumor remission and mild toxicities of half dose of pembrolizumab in this case. In conclusion, the clinical utilization of immunotherapy is an encouraging therapeutic alternative for advanced thymomas. At the same time, patients have to be monitored very carefully, because of the risk to develop irAEs.

## Introduction

Immune checkpoint inhibitor (ICI) therapy has become a promising option for the treatment of late-stage thymic epithelial tumors (TETs) (1). Thymus is a lymphatic system organ for the development of the immune system, which might contribute to high rates of immune-therapy related toxicity events (irAEs) induced by ICI therapy (2). The use of ICI should be carefully considered and closely monitored in this rare thoracic tumor. Herein, we presented a patient with metastatic thymoma administrated of a single dose of anti-programmed cell death protein 1 (PD-1) antibody pembrolizumab, resulting in a storm of irAEs but marked tumor regression.

## Case Description

**Figure 1:**
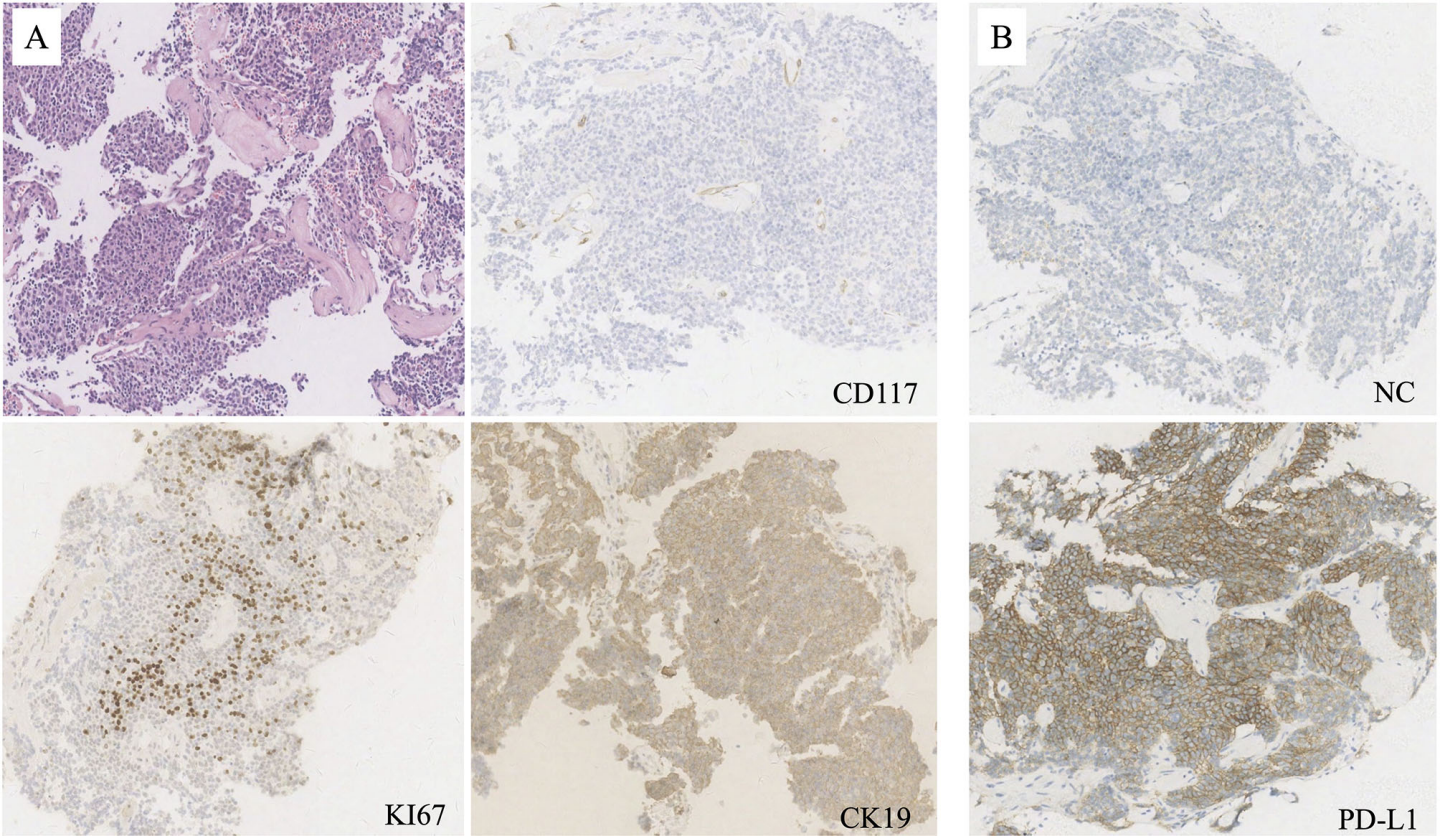
**(A)** Mediastinal needle biopsy indicated type WHO type B3 thymoma (hematoxylin and eosin; original magnification, ×50), with negative staing of CD117, but positive sating of KI67 and CK19 (immunohitochemistry; original magnification, ×50). **(B)** PD-L1 was strongly expressed by 80% of all neoplastic thymic epithelial cells (immunohitochemistry; original magnification, ×50).

**Figure 2:**
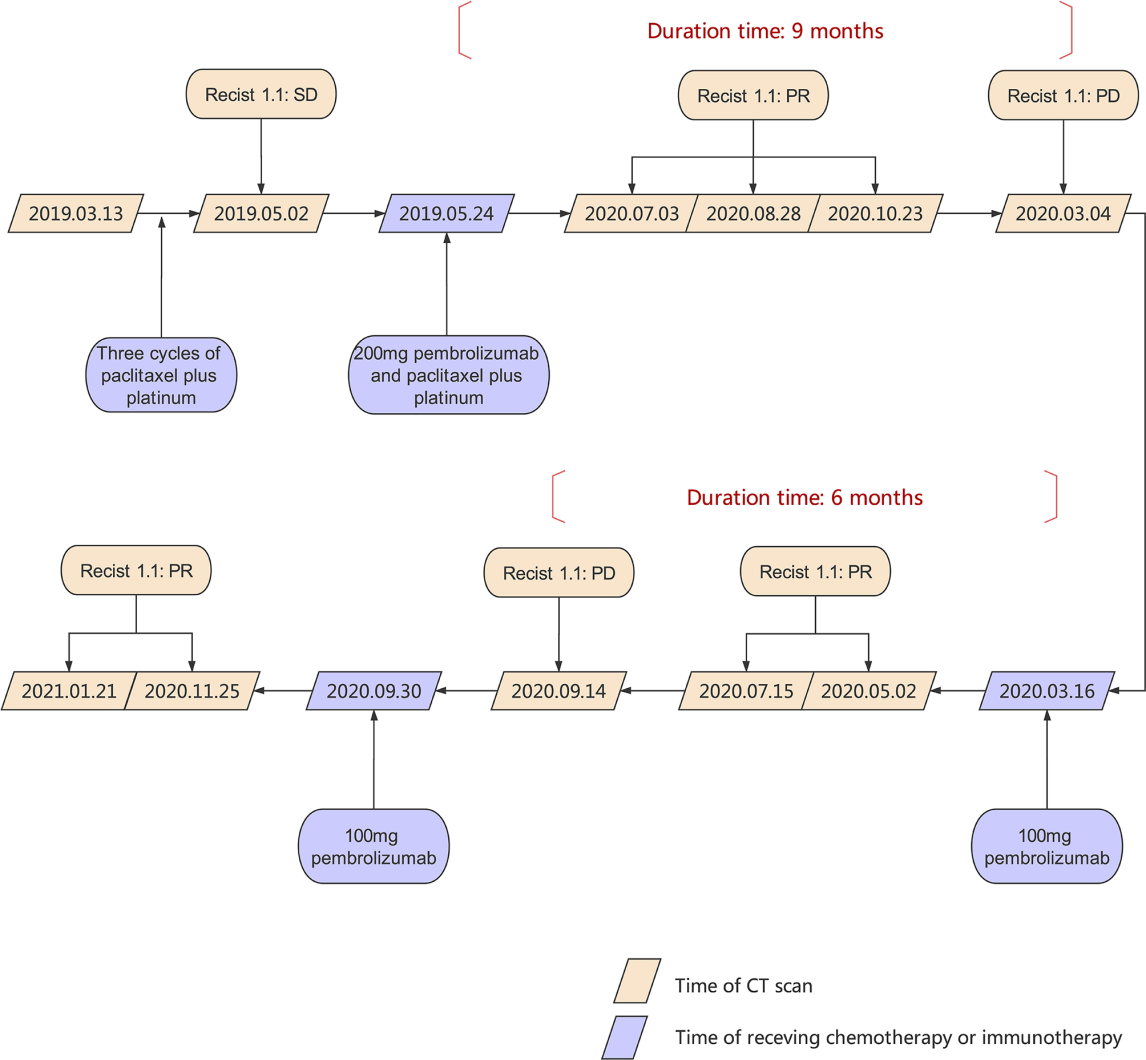
Timeline scheme of major clinical event of the patient since diagnosis.

In March 2019, a 53 year-old woman was diagnosed at our institution as WHO type B3 thymoma with pleural dissemination (**Figure 1A**), and her clinical stage was Masaoka stage IVA. She complained of a dry paroxysmal cough, with no other symptoms. Initially, the patient underwent three cycles of paclitaxel and platinum chemotherapy, and had stable disease. Immunohistochemistry showed 80% expression of PD-L1 in neoplastic thymic epithelial cells (**Figure 1B**). On May 24, 2019, the patient received 200 mg of pembrolizumab, combined with paclitaxel and platinum (**Figure 2**).

**Figure 3:**
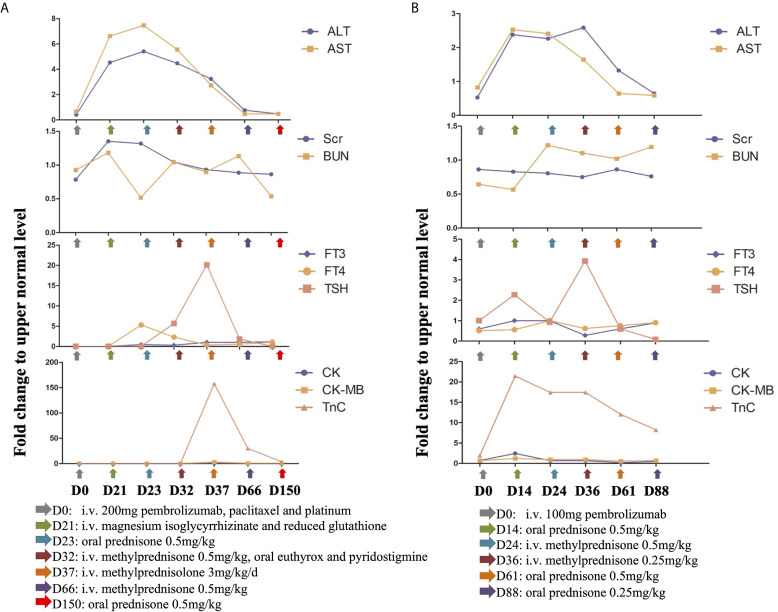
**(A)** Mild irAEs occurred three weeks (D21) post the administration of 200 mg pembrolizumab, which includes grade 2 to 3 liver abnormality, grade 1 kidney dysfunction, grade 1 hypothyroidism and grade 2 myocarditis. Steroids were tapered slowly over a 5-month (D150) period with gradual recovery. **(B)** Grade 1 liver and kidney deficiencies, grade 1 hypothyroidism and grade 2 myocarditis presented as a consequence of 100 mg pembrolizumab (D14). The irAEs reversed following immunotherapy discontinuation and steroids treatment. (Fold change was calculated as a ratio relative to upper limit of normal).

Three weeks later, the patient presented with cough and chest congestion, muscle weakness and fatigability. The blood test indicated liver (grade 2-3) and kidney (grade 1) dysfunction, with ALT of 154 U/L, AST of 225 U/L, urea nitrogen of 8.50 mmol/L, and creatinine of 119 µmol/L (**Figure 3A**). Magnesium isoglycyrrhizinate and reduced glutathione injections didn’t relieve clinical symptoms and dysfunction of liver and kidney. It manifested as irAEs. The patient started the oral administration of prednisone at a dose of 0.5 mg/kg daily. Nine days later, the patient underwent drooping eyelids. Blood analyses indicated a slight decrease of ALT (152 U/L), AST (189 U/L), urea nitrogen (7.51 mmol/L) and creatinine (92 µmol/L), but mild hypothyroidism (grade 1) with TSH of 27.92 miu/L, and FT4 of 7.34 pmol/L (**Figure 3A**). Therefore, the patient was injected 40 mg methylprednisolone daily, and took 50 euthyrox once and 60 mg pyridostigmine three times daily.

After 5 days of therapy, the symptoms were relieved and liver enzymes and kidney function markers turned to normal. However, the patient experienced chest congestion and bradycardia at night. She was free of any heart disease and corresponding symptoms before this happens. Besides, Troponin T (2.210 ng/ml), creatine kinase (731 U/L) and creatine kinase-MB (72 U/L) was increased (**Figure 3A**). Electrocardiogram demonstrated complete right bundle branch block and T-wave inversion. Although endomyocardial biopsy was not performed, it manifested as autoimmune myocarditis (grade 2) according to the presence of typical clinical and laboratory features. Methylprednisolone pulse therapy was given to the patient at a dose of 200 mg daily. The dose of methylprednisolone was gradually reduced according to the levels of serum myocardial enzymes, and maintained at 40 mg daily. The clinical symptoms disappeared soon, and by the end of July 2019, serum myocardial enzyme is nearly back to normal (**Figure 3A**). Subsequently, the patient was discharged from the hospital, followed by oral prednisone, euthyrox and pyridostigmine.

**Figure 4:**
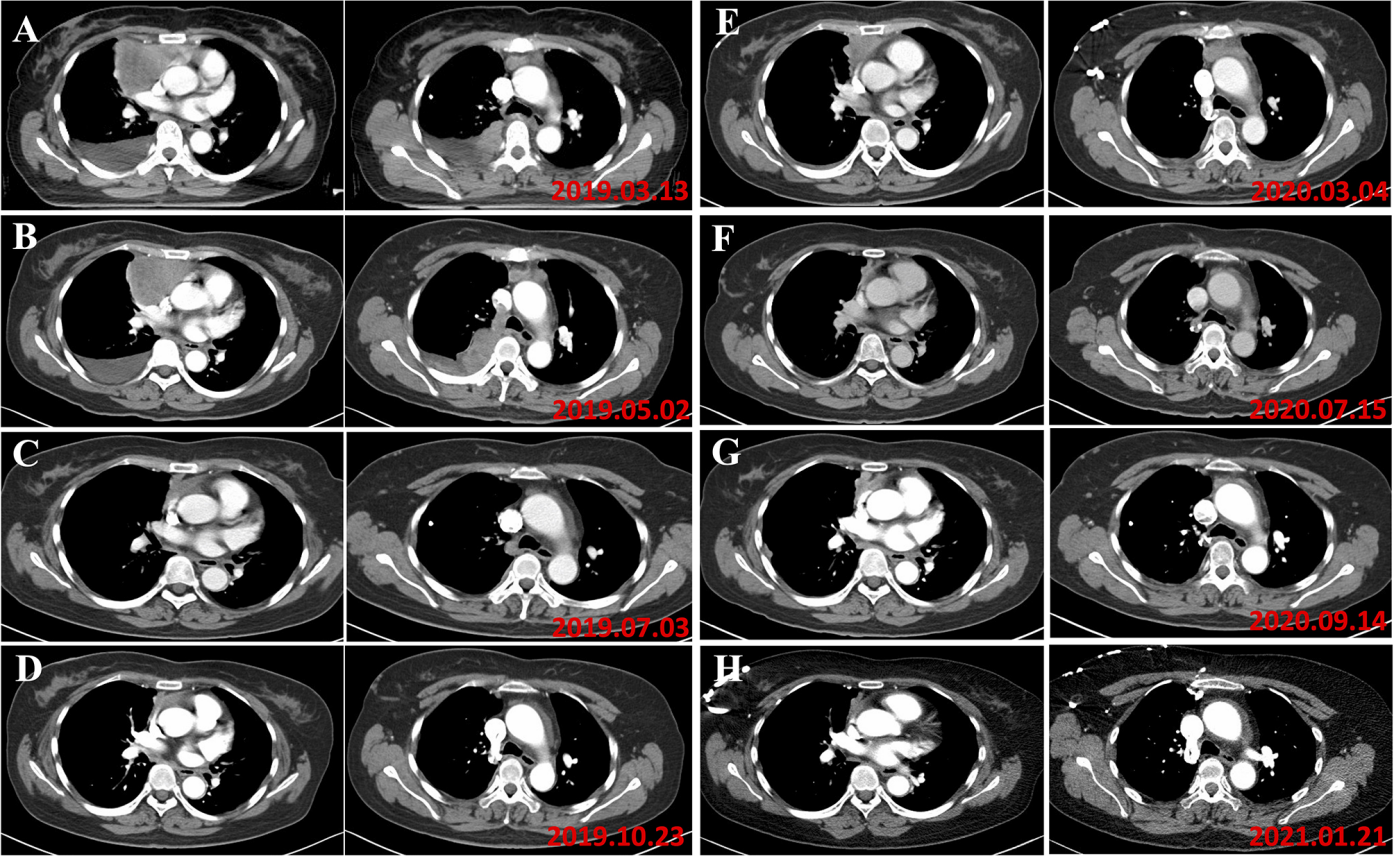
**(A)** In March 2019, the patient was diagnosed as type B3 thymoma with pleural dissemination, and her clinical stage was Masaoka stage IVA. **(B)** In May 2019, the evaluation of tumor response to chemotherapy is SD (RECIST). **(C)** In August 2019, the tumor responded to pembrolizumab. **(D)** In October 2019, a near complete response was achieved following a dose of pembrolizumab. **(E)** In March 2020, the disease progressed and 100 mg of pembrolizumab was administered. **(F)** The patient responded to 100 mg pembrolizumab with partial regression. **(G)** In September 2020, progression of the disease was found. **(H)** The patient responded to 100 mg pembrolizumab again.

The patient was monitored in the outpatient for blood tests every one week at first, and gradually reduced to 1-month interval. At the same time, the patient underwent CT scans every 2 months. A rapid and dramatic response to one dose of pembrolizumab was observed and a near complete response (CR) was achieved by the end of October 2019 (**Figures 4A–D**).

However, at the beginning of March 2020, the patient suffered a dry paroxysmal cough again, and CT scan indicated disease progression (**Figure 4E**). Considering the potential benefit and risk of pembrolizumab, the patient was administered only 100 mg of pembrolizumab, and she presented with grade 1 liver and kidney dysfunction, grade 1 hypothyroidism and grade 2 myocarditis. The patient was administered with steroid therapy, and blood levels gradually returned to normal approximately two months later (**Figure 3B**). Excitedly, she responded to pembrolizumab again with partial regression (**Figures 4F, G**). However, in September 2020, the disease progressed again. One hundred mg of pembrolizumab was administered to patient and the disease partially regressed (**Figure 4H**). Similarly, mild liver dysfunction and myocarditis was successfully managed by steroid therapy.

## Discussion

Thymomas and thymic carcinomas are rare neoplasms that arise from the thymic epithelium. Although rarity of the disease precludes large clinical trials, some phase II clinical trials (2, 3) and case reports (4, 5) have demonstrated the efficacy of immunotherapy for treating advanced-stage TETs. The anti-PD-1 antibody, pembrolizumab, achieved a promising response rate of 22.5% and median progression-free survival of 4.2 months in thymic carcinoma in a single-arm phase 2 study (2). In another Korean single-center phase II study (3), 28.6% of patients with thymoma had partial response. Pembrolizumab is a promisingly therapeutic alternative for refractory TETs when first-line treatment doesn’t work.

Besides, anti-PD-1 therapy can induce durable long-term response in a subset of patients (6). In a retrospective study of 396 melanoma patients receiving single-agent anti-PD-1 therapy, 83.3% of patients who achieved CR remained in remission at 3 years after discontinuation of PD-1 (7). In the case we presented, pembrolizumab was effective in metastatic thymoma. What’s more amazing is that the response maintained 9 months after discontinuation of one cycle of pembrolizumab. To the best of our knowledge, it’s the first reported case with sustained disease control to only one dose of pembrolizumab in metastatic thymoma, implying the significant role of anti-PD-1 in thymoma management.

PD-1 is an immune checkpoint receptor expressed on activated T cells, and it’s also vital in peripheral tolerance and prevention of autoimmunity (8). Therefore, it’s not surprising to observe irAEs induced by PD-1 antibody. The most commonly observed are rash, colitis, pneumonitis, hepatitis, nephritis, and endocrinopathies (8). In the Keynote-002 study, 57%-60% of grade 1 to 2 irAEs and 14% of grade 3 to 4 irAEs were reported in metastatic melanoma. Due to the thymic origin, the rates of irAEs are much higher in TETs than other cancer types, especially myocarditis and myasthenia gravis (9, 10). Besides, compared with thymic carcinoma, thymomas often present with autoimmune diseases linked to T-cell-mediated autoimmunity, particularly myasthenia gravis (11, 12). Furthermore, the rates of irAEs are much higher in thymomas than thymic carcinoma following pembrolizumab treatment. In the Korean study consisting of 26 thymic carcinoma and 7 thymoma and patients (3), 15.4% of thymic carcinoma patients developed severe irAEs, while it’s 71.6% in thymoma patients.

The myocarditis is rare among the irAEs, but it’s often life-threatening, accounting for up to 22% of death associated with anti-PD-1 toxicities (13, 14). The underlying mechanism of ICI-associated myocarditis is not completely understood. It was reported that the shared antigen between the tumor and myocardium, and T cell-mediated immunity might contribute (15, 16). Careful monitoring and active follow-up strategies are essential, such as blood test and ultrasound, to prevent and detect early signs of myocarditis and other irAEs. For example, special care should be taken when administering pembrolizumab to patients with reported risk factors for ICI-associated myocarditis, who received combined therapy of anti-CTLA-4 and anti-PD-1, who have history of heart disease, or who underwent previous chest radiotherapy (17). Baseline and dynamic electrocardiogram, serum myocardial enzymes and cardiorespiratory symptoms should be monitored (16). Ma et al. (18) also reported that elderly patients and male patients were more likely to develop ICIs-related myocarditis. In future study, it’s of utmost significance to explore markers to predict irAEs and alert physicians to avoid delaying the start of appropriate treatment, and ultimately identify those who will benefit from pembrolizumab without severe irAEs (16).

The majority of the irAEs, although severe in some cases, is manageable and reversible following immunotherapy discontinuation and steroids treatment. Steriods will not affect the efficacy of immunotherapy (8). In the case we presented, grade 2 myocarditis was observed during low dose of methylprednisolone treatment, and relieved following methylprednisolone pulse therapy. Patients are at risk for developing irAEs again on rechallenge with ICIs, and the recurrence rate of any grade irAE ranged from 25% to 50% (19, 20). The extent to which irAEs will limit deployment of ICIs is still under doubt. Generally, for patients experiencing severe irAEs, permanent discontinuation of ICIs is recommended (19). For cases with low-grade irAEs, an ICI rechallenge after temporary discontinuation appears conceivable (19). Rechallenge with ICI therapy is still a difficult decision because of the limited available study regarding the safety of rechallenge of ICIs after the presence of irAEs, and absence of formal guidelines (20). Therefore, for ICIs resumption, we have to take the original irAEs in careful consideration, and balance the clinical benefit of rechallenge and the potential risk of severe irAEs.

Higher incidence of irAEs has been observed with the higher dosage of PD-1 antibodies (9). Therefore, it’s necessary to optimize dose to minimize the irAEs induced by PD-1 antibody while maintaining clinical effectiveness. Clinical efficacy of pembrolizumab at a fixed dose of 100 mg 3-weekly was also reported, with no difference in progression-free survival and overall survival with 200 mg pembrolizumab (21). Although no guidelines recommend dose reduction of immunotherapy re-treating following initial irAEs (8), we observe remarkable tumor remission and mild toxicities of half dose of pembrolizumab in this case we presented. Overall, rechallenge of pembrolizumab require further investigation in well-designed prospective randomized trials, especially for those with lethal myocarditis.

In conclusion, the clinical utilization of immunotherapy is an encouraging therapeutic alternative for advanced thymomas refractory to platinum chemotherapy. At the same time, patients have to be monitored very carefully, because of the risk to develop irAEs.

## Data Availability Statement

The raw data supporting the conclusions of this article will be made available by the authors, without undue reservation.

## Ethics Statement

The studies involving human participants were reviewed and approved by The Second Affiliated Hospital, Zhejiang University School of Medicine. The patients/participants provided their written informed consent to participate in this study. Written informed consent was obtained from the individual(s) for the publication of any potentially identifiable images or data included in this article.

## Author Contributions

QW designed the study. LS and HC collected and analyzed patient data. HC wrote the manuscript, and QW did the modification. All authors contributed to the article and approved the submitted version.

## Funding

This work was partly supported by grants from National Natural Science Foundation of China (82073332), China Postdoctoral Science Foundation (519000-X91919), Zhejiang Provincial Natural Science Foundation of China (LQ21H160035), CSCO-Roche research funding (Y-Roche2019/2-0088), and the Medical Health Science and Technology Project of the Health Commission of Zhejiang province (2020381184).

## Conflict of Interest

The authors declare that the research was conducted in the absence of any commercial or financial relationships that could be construed as a potential conflict of interest.
